# Public health round-up

**DOI:** 10.2471/BLT.19.011119

**Published:** 2019-11-01

**Wild poliovirus incidence rising**

A young girl is vaccinated against polio in one of the WHO/UNICEF-supported vaccination posts along the border between Afghanistan and Pakistan.

**Figure:**
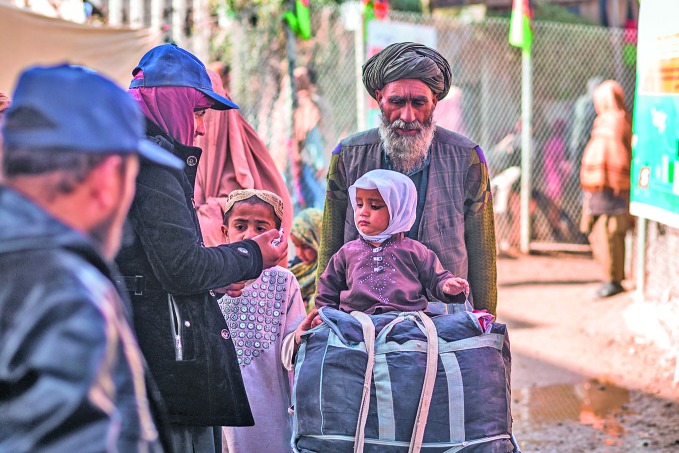
UNICEF/Jim Huylebroek

## Syrian health crisis

Up to 200 000 people have been displaced following the recent increase in military operations in the Syrian Arab Republic. As of 13 October, an estimated 1.5 million people needed health aid.

Health services in the northeast of the country have been severely impacted, including the national hospital in Ras Al-Ain and the national hospital and two health centers in Tel Abyad, all which were out of service, as of 13 October. Widespread health worker shortages are aggravating an already critical situation.

The World Health Organization (WHO) and health partners are working hard to respond to urgent health needs, positioning medical supplies in Qamishly, a city on the border with Turkey. Some people requiring hospitalization have been referred to a WHO-supported facility in Al-Hassakeh, and WHO is in the process of contracting two additional hospitals in Al-Hassakeh and Al-Raqqa to support referral services.

WHO calls on all parties to the conflict to comply with international humanitarian law to protect all civilians, including health care workers and patients, as well as health facilities.

https://reliefweb.int/report/syrian-arab-republic/who-gravely-concerned-about-humanitarian-situation-northeast-syria

## Polio concern

The Emergency Committee under the International Health Regulations (2005), convened by WHO on 16 September 2019 reported that 73 people have been infected with wild poliovirus in the year to date, compared to 15 for the same period in 2018, with most of the increase due to ongoing outbreaks in Pakistan.

The committee expressed concern regarding the trend and announced that the risk of international spread of wild poliovirus is at its highest since 2014 when a public health emergency of international concern was first declared.

The committee also noted a high risk of outbreaks of vaccine-derived poliovirus infections in other countries.

https://www.who.int/news-room/detail/03-10-2019-statement-of-the-twenty-second-ihr-emergency-committee-regarding-the-international-spread-of-poliovirus

## Immunization challenges

The Strategic Advisory Group of Experts (SAGE) on Immunization met on 8-10 October. Among the topics discussed were measles and rubella elimination, vaccine supply and access to the human papillomavirus vaccine, polio eradication, clinical trials of a novel type 2 oral polio vaccine, interim Ebola vaccine recommendations; and a new global strategy for immunization.

Headline topics included diphtheria-tetanus-pertussis coverage (coverage stalled at 86% globally, Africa having the highest proportion of unreached children) and inequity in measles vaccination coverage. The group noted WHO’s engagement with social media platforms, which has resulted in changes in the algorithms that direct users to information on vaccines.

SAGE also expressed serious concern regarding polio eradication efforts, notably the upsurge in wild poliovirus cases detected in Afghanistan and Pakistan and the inability of the eradication programme to effectively control outbreaks of circulating vaccine-derived polioviruses in Africa and Asia.

https://www.who.int/immunization/sage/meetings/2019/october/SAGE_Oct_2019_Meeting_Highlights.pdf?ua=1

## Declaration on universal health coverage

World leaders adopted a declaration on universal health coverage at United Nations headquarters in New York on 24 September.

Dr Tedros stated that the declaration represents a landmark for global health and development. “The world has 11 years left to make good on its sustainable development goals. Universal health coverage is key to ensuring that happens,” he said.

On the eve of the meeting WHO launched its *Universal health coverage global monitoring report 2019*. The report calls for governments to increase spending on primary healthcare by at least 1% of their gross domestic product to meet health coverage targets agreed in 2015.

https://www.who.int/news-room/detail/23-09-2019-who-welcomes-landmark-un-declaration-on-universal-health-coverage

## Agencies support health SDGs

A group of twelve multilateral agencies, accounting for nearly one-third of all development assistance to health, launched a joint plan to better support countries over the next 10 years to accelerate progress towards the health-related sustainable development goals.

https://www.who.int/news-room/detail/24-09-2019-multilateral-agencies-launch-a-joint-plan-to-boost-global-health-goals

## Mistreatment of women during childbirth

New research assessing the scale of mistreatment of women during childbirth in health facilities in Ghana, Guinea, Myanmar and Nigeria revealed that more than one in three women experience physical or verbal abuse from healthcare staff.

The WHO-led research, which includes continuous observations of women in labour, was published in the *Lancet* on 9 October. It reveals that 838 (42%) of the 2016 women observed were mistreated, 14% experiencing physical abuse – including slapping and punching, 38% experiencing verbal abuse – including scolding and mocking. The research also found high incidences of non-consensual caesarean sections, episiotomies and vaginal examinations.

https://www.thelancet.com/journals/lancet/article/PIIS0140-6736(19)31992-0/fulltext

## Sudan cholera campaign

Sudan launched an oral cholera vaccination campaign on 11 October in response to the ongoing cholera outbreak which was declared on 8 September. More than 1.6 million adults and children over the age of one year were vaccinated in Blue Nile and Sinnar states.

The first round of the campaign was scheduled to conclude on 16 October and is to be followed by a second round in November or December to ensure people are protected for at least the next three years.

As of 9 October, 262 people were suspected as having cholera and eight cholera-related deaths had been reported in the Blue Nile and Sinnar states.

https://www.unicef.org/sudan/press-releases/vaccination-campaign-against-cholera-kicks-sudan

**Cover photo**

WHO staff collect mosquitos in Puerto Princesa, Palawan island, Philippines. As of 21 September 2019, the cumulative number of dengue infections reported in the Philippines was 322 693, more than double the 149 849 infections reported during the same period in 2018.

**Figure:**
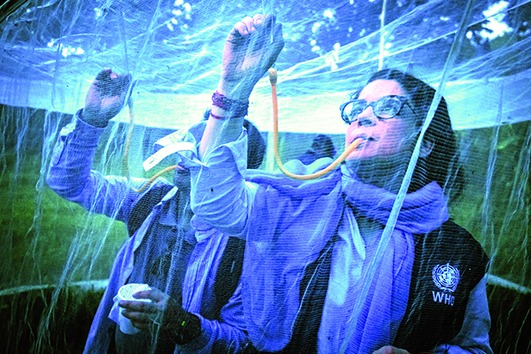
WHO/Yoshi Shimizu

## Decrease in alcohol consumption, Russian Federation 

Annual alcohol consumption in the Russian Federation dropped between 2003 and 2016, falling from 20.4 litres per capita of population aged over 15 years to 11.7 litres, according to *Alcohol policy impact case study: the effects of alcohol control measures on mortality and life expectancy in the Russian Federation*, a WHO report published on 1 October.

The decline follows implementation of comprehensive alcohol control measures by the government which started in 2003. The trend mirrored an increase in life expectancy, which reached a historic high in 2018, at almost 68 years for men and 78 years for women.

https://apps.who.int/iris/bitstream/handle/10665/328167/9789289054379-eng.pdf?sequence=1&isAllowed=y

## Ebola challenges shift

The number of people infected with Ebola virus disease is declining, and there is a shift in infection hot spots from urban settings to rural, harder-to-reach communities. Difficulties accessing some remote areas and the relatively lower awareness of Ebola in these settings is complicating the response. The security situation continues to be a matter for concern, with ongoing conflict involving multiple armed groups.

There were 14 new, confirmed cases reported during the week of 30 September through 6 October. This is down from 126 cases per week at peak transmission in April 2019. As of 8 October, a total of 3207 Ebola infections had been reported, and 2144 people had died.

The outbreak, declared on 1 August, 2018, started in North Kivu and has since spread to parts of Ituri and South Kivu provinces.

https://www.who.int/csr/don/10-october-2019-ebola-drc/en/

**Looking ahead**

7 – 9 November. Leading Minds for Children and Young People conference. Florence, Italy.

16 – 17 November. Global Ministerial Conference on Ending TB in the SDG Era. Moscow, Russian Federation.

19 – 20 November. Reaching the Last Mile Forum. Abu Dhabi, United Arab Emirates.

19 – 21 November. Meeting of the Parties to the Protocol on Water and Health. Belgrade, Serbia.

